# Effects of Temperature and Salinity on the Germination of *Triticum aestivum*

**DOI:** 10.1021/acs.jchemed.6c00286

**Published:** 2026-07-20

**Authors:** Cecilia Martínez-Castillo, Manuel Arias-Estévez, Manuel Conde-Cid, Daniel Arenas-Lago, Antía Gómez-Armesto

**Affiliations:** † Department of Plant Biology and Soil Science, Faculty of Sciences, 16784 University of Vigo, As Lagoas S/N, 32004 Ourense, Spain; ‡ REQUIMTE/LAQV, ISEP, Polytechnic of Porto, Rua Dr. António Bernardino de Almeida 431, 4249-015 Porto, Portugal

**Keywords:** Upper-Division Undergraduate, Graduate Education/Research, Environmental Chemistry, Agricultural Chemistry, Laboratory Instruction

## Abstract

In this laboratory exercise, students explore the potential impacts of temperature and salinity on agricultural systems. Specifically, wheat seed germination was evaluated across a temperature range of 15–35 °C and in the presence or absence of sodium chloride (NaCl), thereby simulating scenarios of rising temperatures and increasing soil salinity. The primary objective of this activity was to raise students’ awareness of the challenges that climate change may pose to crop production and, consequently, to promote critical thinking about agronomic strategies that could mitigate these adverse effects. The results indicate that both root and shoot growth of wheat seedlings increase as temperature rises within the tested range. However, at elevated temperatures (35 °C), particularly under saline conditions, both root and shoot development were markedly reduced. These findings allow students to examine the interactive effects of temperature and salinity stress while connecting experimental observations to broader environmental and agricultural issues.

## Graphical abstract

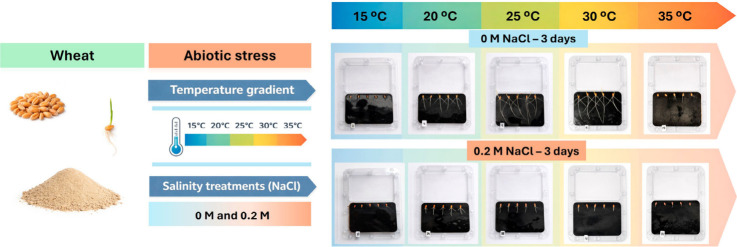

## Introduction

According to FAO data, the global population is projected to reach 9.1 billion by 2050, representing an increase of approximately one-third compared to the current population. As a result, agricultural production must increase and food security must be strengthened to meet the needs of this growing population. However, agricultural output has not kept pace with population growth, primarily due to both biotic and abiotic stresses affecting soils, as well as environmental pollution.^1^

Climate change is likely to exacerbate these challenges, mainly through rising temperatures. According to the United Nations, the Earth’s average temperature is now 1.1 °C higher than it was at the end of the 19th century. The past decade (2011–2020) was the warmest on record, and each of the past four decades has been warmer than any decade since 1850. Rising temperatures are among the most concerning stresses for crops, negatively affecting growth, germination rates, and photosynthetic activity, and, in some cases, causing plant mortality.^2–4^ Increased temperatures also amplify other stresses, such as soil salinity (under conditions of water scarcity), which tends to accumulate in the upper layers of the soil. This salinity can also be increased by other activities, such as the use of saline water from industrial or mining activities. Salinity produces three main effects on crops: increased osmotic pressure, nutrient imbalance, and elemental toxicity, resulting in reduced yield and greater susceptibility to disease. At the cellular level, salinity can damage cell membranes, increase reactive oxygen species production, and decrease photosynthetic and enzymatic activity.^5^ Saline soils often exhibit poor porosity and are prone to waterlogging, leading to limited water and nutrient availability.

The initial effects of both temperature and salinity are manifested during seed germination. This process is important not only for crop productivity but also for environmental conservation, as improved germination and seedling establishment can help prevent severe soil erosion by increasing vegetative cover.

Wheat was selected for this experiment because it is the most widely cultivated crop in the world in terms of cultivated area (219 million ha)^6^ and is relatively tolerant to salinity. However, the combined effects of high temperature and salinity on germination are less well understood and may have antagonistic effects, potentially reducing germination rates. Therefore, these interactions should be considered when selecting suitable cultivation sites, choosing soil remediation techniques, planning recultivation strategies, and selecting different species.

Phytotoxicity assays, such as the Phytotoxkit, have proven highly effective for both educational and research purposes in studying germination under stress conditions.^7^ We propose that these assays can also be used to investigate potential abiotic stresses on crops caused by the combined effects of elevated temperature and salinity driven by climate change. The Phytotoxkit system offers several advantages over conventional phytotoxicity assays, including the ability to perform multiple tests simultaneously, vertical incubation of plates to minimize storage space, and the option to delay analysis and measurements using digital images of the plates. From an educational perspective, these assays enable the development of key competencies such as experimental design, variable control, data analysis, and critical interpretation within an applied and realistic biological framework. Furthermore, they facilitate the understanding of concepts such as interactions between environmental factors, the existence of tolerance thresholds, and nonlinear organism responses, which are central to soil and environmental sciences. The combination of variables such as salinity and temperature introduces an additional level of complexity that enhances scientific reasoning and learning, which has been shown to be particularly effective in experimental science education.^8^

Compared to more simplified methodologies such as Petri dish assays,^9^ which are widely used due to their simplicity and rapid implementation, phytotoxicity assays provide a context that is closer to real-world conditions, thereby improving students’ ability to transfer acquired knowledge to agricultural and environmental scenarios. It has been demonstrated that the outcomes of germination and growth tests significantly depend on the substrate used, as factors such as adsorption or nonhomogeneous contaminant distribution may alter plant responses in simplified systems such as filter paper in Petri dishes.^10^ Therefore, these assays not only support knowledge acquisition but also promote the development of practical skills and critical thinking, which are essential components of scientific training.

This laboratory exercise was conducted with second-year students enrolled in Agricultural Engineering and Environmental Science programs. It examines the influence of temperature on the germination of wheat, a globally important crop. The primary objectives were to increase students’ understanding of the potential adverse effects of climate change on agricultural production and to foster environmental awareness that promotes sustainable agricultural practices under conditions of rising temperature and salinity, highlighting germination as the first step toward achieving satisfactory productivity. From a pedagogical point of view, it is intended that students acquire competencies in soil productivity and environmentally friendly agricultural practices; specifically, the following objectives are proposed:

(i) that students understand how environmental factors such as salinity and temperature influence germination and initial plant growth, as well as the concept of abiotic stress in agricultural systems;
(ii) that students develop skills to analyze and interpret experimental data obtained in phytotoxicity tests;
(iii) that students identify patterns in plant responses to stress conditions; and
(iv) that students are able to relate experimental results to real problems associated with climate change and agricultural production.

To support these objectives, students were provided with structured pre-laboratory materials and guiding questions linking chemical concepts to experimental observations. From a chemistry education perspective, this activity allows students to apply key concepts such as osmotic potential, ionic strength, ion toxicity, and temperature-dependent biochemical processes to a real-world environmental problem. In this way, students connect fundamental chemical principles with observable biological responses under stress conditions.

This study is designed for undergraduate students studying Soil Science in degree programs related to agriculture, environmental engineering, and environmental sciences. Although the activity was implemented with second-year undergraduate students, it can be adapted to different educational levels, depending on the depth of data analysis and discussion.

## Experimental Section

### Material and Methods

Phytotoxicity assays (Phytotoxkit) were conducted following the procedure recommended by MicroBioTests, Inc. (Microbiotests Toxkit) (Phytotoxkit, 2004), using *Triticum aestivum* (common wheat) as the model species. Transparent plastic plates included in the Phytotoxkit system were used to observe seed germination and seedling growth, allowing the measurement of shoot and root lengths at the end of the assay (after 3 days of incubation). During this incubation period, students carried out other more traditional soil science practices such as determining pH, soil texture, effective cation exchange capacity, and soil organic matter content (variables that can be seen in Table 1).

**Table 1:** Soil Characteristics Used in the Germination Experiments^a^

Soil characteristic^a^	Value
pH	9.0
Organic matter (%)	1.0
Sand (%)	70.0
Silt (%)	24.5
Clay (%)	5.5
Ca_e_ (cmol_c_ kg^–1^)	23.9
Mg_e_ (cmol_c_ kg^–1^)	1.0
K_e_ (cmol_c_ kg^–1^)	0.1
Na_e_ (cmol_c_ kg^–1^)	0.2
Al_e_ (cmol_c_ kg^–1^)	0.1
eCEC^b^ (cmol_c_ kg^–1^)	25.3

a Data provided by the instructors.

b Ca_e_, Mg_e_, K_e_, Na_e_, and Al_e_ refer to the exchangeable contents of calcium, magnesium, potassium, sodium, and aluminum, respectively.

c eCEC refers to the effective cation exchange capacity.

The experiments were performed using a sandy loam soil with the following characteristics: pH 9.0, 70.0% sand (2–0.05 mm), 24.5% silt (0.05–0.002 mm), and 5.5% clay (<0.002 mm). The soil contained 1.0% organic matter and exhibited a low cation exchange capacity (Table 1). A detailed description of the methods is provided in subsection S1.1.1 of the Supporting Information.

### Procedure Phases

During the incubation phase, the influence of temperature on seed germination and early seedling growth was evaluated by incubating the samples at 15, 20, 25, 30, and 35 °C. In parallel, salinity experiments were conducted by incubating the same number of plates at the same temperatures using a sodium chloride (NaCl) solution at a concentration of 0.2 M. Previous experiments carried out by the instructors at different NaCl concentrations (0, 0.01, 0.1, and 0.2 M) indicated that both root and shoot growth were more visible and therefore more educational at a concentration of 0.2 M (see Table S1 in the Supporting Information). The objective was to analyze the physiological response of the seeds under different thermal conditions and high-salinity conditions. Both factors represent potential scenarios associated with climate change, as rising soil temperatures increase evaporation and promote soil salinization.

(a) *Determination of water volume per soil sample*: The volume of water required for each soil sample was calculated by adding 50 mL of water to 90 cm^3^ of soil. Once the soil was saturated, a cylinder equipped with a porous membrane was placed on its surface, and excess water was removed with a Pasteur pipet through the membrane until the soil reached its maximum water retention capacity. The difference between the volume of water added (50 mL) and the volume removed was used to determine the amount of water necessary to maintain each soil sample in a saturated state during the phytotoxicity assays.
(b) *Soil preparation*: One hundred grams of each soil sample were placed and evenly spread on the bottom of transparent plates. The soil was leveled, and the appropriate volume of water or NaCl solution, as calculated in step (a), was added.
(c) *Seed placement and incubation*: The soil was covered with a black filter. Five wheat seeds were evenly placed on the filter in each plate. The plates were then closed, positioned vertically, and incubated in the dark for 3 days at 15, 20, 25, 30, and 35 °C in climatic chambers (Velp Scientifica FOC 200IL, Monza, Italy). After the incubation period, seeds with a radicle longer than 1 mm were considered germinated.
(d) *Image analysis and index calculation*: Plates were photographed, and the resulting images were analyzed using ImageJ software (version 1.52p, National Institutes of Health, Bethesda, MD, USA). Root length and shoot length data were obtained for all germinated seeds. These measurements were used to determine the percentage of germinated seeds (G%), mean root length, and mean shoot length. Details of the germination test are described in subsection S1.1.2 of the Supporting Information.

The Germination Index (*G*_Index_) and the Shoot Influence Index (*P*_Index_) for the species under study were also calculated.^11^

*G*_Index_ was calculated using eq 1:

$$G_{\mathrm{Index}}\;\left(\%\right)=\frac{G_{S}\cdot L_{S}}{G_{C}\cdot L_{C}}\times 100$$ 1

where *G*_*S*_ and *L*_*S*_ are the germination percentage and average root length (mm), respectively, and *G*_*C*_ and *L*_*c*_ are the corresponding values for seedlings in the control plate (20 °C, distilled water).

*G*_Index_ values were interpreted as follows:^8^

- *G*_Index_ = 90%–110%: normal growth compared to the control.
- *G*_Index_ < 90%: inhibitory effect compared to the control.
- *G*_Index_ > 110%: stimulatory effect compared to the control.

Similarly, *P*_Index_ was calculated using eq 2:

$$P_{\mathrm{Index}}\;\left(\%\right)=\frac{G_{S}.{\mathrm{Pa}}_{S}}{G_{C}.{\mathrm{Pa}}_{C}}\times 100$$ 2

where *G*_*S*_ and *Pa*_*S*_ are the germination percentage and average shoot length (mm), respectively, and *G*_*C*_ and *Pa*_*C*_ are the corresponding values for seedlings in the control soil (20 °C, distilled water).

*P*_Index_ values were interpreted as follows:

*P*_Index_ = 90%–110%: normal shoot growth compared to the control.
*P*_Index_ < 90%: inhibitory effect on shoot growth compared to the control.
*P*_Index_ > 110%: stimulatory effect on shoot growth compared to the control.

### Educational Innovation and Pedagogical Significance

This laboratory activity introduces an interdisciplinary approach that integrates environmental chemistry principles with plant physiological responses through a controlled experiment simulating climate change scenarios. Unlike conventional germination assays, the activity explicitly connects key chemical concepts, such as osmotic potential, ionic strength, ion toxicity, and temperature-dependent processes, with measurable biological responses, including germination percentage, root length, and shoot development.

The design promotes higher-order learning by engaging students in (i) quantitative data acquisition and analysis through digital image processing (ImageJ), (ii) the calculation and interpretation of germination and growth indices (*G*_Index_ and *P*_Index_), and (iii) the evaluation of the combined effects of temperature and salinity as interacting stress factors. Through this process, students move beyond descriptive observations and develop mechanistic explanations based on chemical principles, including osmotic pressure, ionic strength, ion-specific toxicity, and water availability under saline conditions. Examples of student responses illustrating these interpretations are provided in the Supporting Information (Section S2).

In addition, the activity promotes active and collaborative learning, as students generate reproducible data sets, compare results across groups, and collectively interpret variability and experimental trends. This approach strengthens key competencies, including data interpretation, quantitative reasoning, and scientific communication, as evidenced by laboratory reports, postlaboratory questionnaires, and assessment activities summarized in the Supporting Information (Tables S5–S7).

By situating chemical concepts within the context of soil salinization and climate change, the activity enables students to link experimental results with real-world agricultural challenges, promoting a deeper understanding of how chemistry underpins crop responses to environmental stress and supporting informed decision-making in sustainable agriculture. This approach is consistent with inquiry-based science education frameworks, which emphasize the application of scientific concepts to authentic problems and promote conceptual understanding through active engagement with experimental evidence.^8^

Unlike conventional germination experiments frequently used in introductory biology or environmental science laboratories, this activity explicitly integrates chemical principles such as osmotic pressure, ionic strength, ion-specific toxicity, and soil chemical properties within a climate-change framework. Furthermore, the activity is embedded within a broader sequence of soil chemistry laboratory exercises, enabling students to connect physicochemical soil properties with plant responses and environmental sustainability challenges. This multidisciplinary integration represents the principal pedagogical innovation of the activity and distinguishes it from more-traditional germination-based laboratory activities.

### Student Work

Before the lab session, students were provided with preparatory materials that included a theoretical background and guiding questions designed to connect chemical concepts, such as osmotic stress, ionic strength, and ionic toxicity, with the experimental conditions (Subsection S1.2.1. of the Supporting Information). After completing the experiment, students were asked to submit a lab report that included data analysis, calculation of germination and growth rates, and interpretation of the results in the context of climate change and agricultural sustainability. In addition, students completed a post-laboratory questionnaire aimed at evaluating their understanding of key concepts and their ability to relate experimental results to climate change and agricultural contexts. The questionnaire is provided in the Supporting Information (Subsection S2). Twelve groups were formed, each consisting of three students (36 students in total). A phytotoxicity assay using the Phytotoxkit was conducted to evaluate the effects of temperature and salinity as stress factors on the germination and early growth of *Triticum aestivum*, reflecting conditions associated with climate change.

Each group prepared a total of 10 phytotoxicity plates (5 wheat seeds per plate): 5 control plates with distilled water and 5 plates with a 0.2 M NaCl solution. These plates were incubated at five different temperatures (15, 20, 25, 30, and 35 °C). In total, across all groups, 120 plates were prepared.

For data analysis of student results, the dataset obtained from the 12 student groups was first inspected for consistency across replicates. Each experimental condition was evaluated across all student groups, resulting in multiple measurements per condition. To improve robustness at the group level and minimize the influence of extreme values commonly observed in educational laboratory datasets, the highest and lowest values for each experimental condition were excluded. The remaining data (*n* = 10 per condition, corresponding to measurements obtained across all groups after exclusion of extremes) were used for graphical representation and subsequent group-level analysis.

Instructor data were obtained from five independent experimental replicates (*n* = 5), each conducted by different instructors following the same experimental protocol under identical conditions.

At the end of the experiment, data were compiled and discussed collectively to draw conclusions from the study. The average time required for students to complete the laboratory exercise was approximately 8 h: 4 h on the first day for plate preparation and 4 additional hours for measuring root and shoot lengths after the three-day incubation. Detailed timing for each phase of the experiment is provided in the Supporting Information (Subsection S1.5). This laboratory activity was integrated into the Soil Science course within the Agricultural Engineering and Environmental Science programs, a compulsory second-year course (6 ECTS) that combines theoretical and practical sessions. The course includes a series of structured laboratory activities distributed throughout the term, focused on soil physicochemical properties, such as texture analysis, organic matter determination, soil pH, cation exchange capacity, and exchangeable cations. The relationships between these complementary laboratory activities, their chemistry-related learning outcomes, and the corresponding assessment approaches are summarized in Table S7 of the Supporting Information.

The present experiment corresponds to one of these laboratory sessions and was conducted over a one-week period within the practical component of the course. Within this framework, the activity complements other laboratory exercises by providing an applied perspective on soil–plant interactions, specifically addressing how environmental factors such as temperature and salinity influence plant development. Students enrolled in this course have already completed a structured foundation in chemistry, biology, and earth sciences during the first year of their program. Specifically, they take introductory courses in general chemistry, in which they develop fundamental knowledge of solution chemistry, concentration, ionic interactions, and laboratory practices, as well as more advanced topics such as acid–base equilibria, solubility, and chemical kinetics.

At the same time, students receive training in basic biological concepts, including cellular processes, metabolism, and plant physiology, and in geosciences, where they acquire knowledge about terrestrial materials and environmental processes. This interdisciplinary training enables students to understand the physicochemical foundations of soil processes and plant responses to environmental stress.

Consequently, although the course is not a traditional chemistry course, students are able to apply fundamental chemical principles—such as osmotic potential, ionic strength, and solute interactions—to interpret experimental results in an environmental and agronomic context.

Although the experimental design focuses on controlled variables such as temperature and salinity, the connection with soil chemistry is reinforced through its integration with other laboratory activities within the course. Students work with the same soil samples across different practical sessions, where they determine key physicochemical properties such as pH, cation exchange capacity, and organic matter content.

This approach allows students to relate the results of the phytotoxicity assay to previously characterized soil properties, facilitating a more comprehensive interpretation of plant responses, in terms of soil chemical conditions. The combined analysis is further discussed during laboratory sessions, promoting the integration of chemical and biological perspectives in the soil–plant system.

While the present activity was designed to maintain simplicity and focus on key variables, this integrated framework provides a solid basis for extending the experiment toward more-detailed soil chemistry analyses in advanced courses.

This integration allows students to connect chemical properties of soils with observable biological responses under controlled experimental conditions, reinforcing the learning outcomes related to data interpretation, critical thinking, and an understanding of soil processes. The total cost of the laboratory activity is presented in Table S3 of the Supporting Information, while the cost per student (estimated at €15–20 per student) is detailed in Subsection S1.6.1 of the Supporting Information.

## Hazards

No significant hazards are associated with this experiment, as the reagents used (0.2 M NaCl and air-dried soil) are of low risk. The characteristics of the NaCl used are presented in Table S2 (Supporting Information). Standard laboratory safety practices are required, including the use of a laboratory coat, safety glasses, and gloves. Direct contact with soil should be avoided by using appropriate tools (e.g., spatulas and forceps), and basic hygiene practices such as handwashing after handling samples are required. Care should be taken to avoid dust generation and cross-contamination between treatments. Waste soil and plant material are considered nonhazardous and must be disposed of according to institutional laboratory guidelines. More details are described in the Supporting Information (Subsection S1.4.1.).

## Results and Discussion

The germination results are shown in Figure 1. From the analysis of Figure 1a, which presents the results obtained by the instructors, it can be observed that germination percentages were 100% in all cases, except for incubations at 30–35 °C and seeds exposed to a 0.2 M NaCl solution at these temperatures. No seeds germinated at 35 °C in the presence of 0.2 M NaCl. The results obtained by the students were very similar, as shown in Figure 1b.

**Figure 1:**
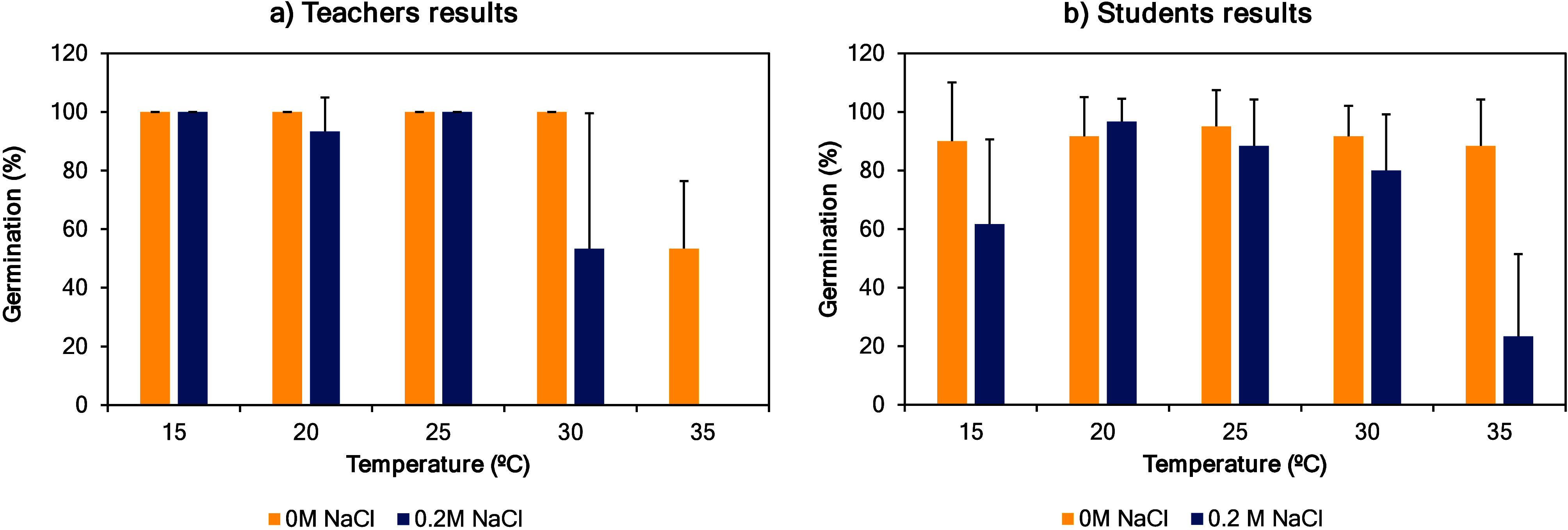
Germination results of *Triticum aestivum* under different temperature (15, 20, 25, 30, 35 °C) and salinity conditions (0 M NaCl and 0.2 M NaCl), expressed as percentage of germination: (a) teachers’ results (*n* = 5) and (b) students’ results (*n* = 10). Error bars indicate 95% confidence intervals.

Root growth results, expressed as *G*_Index_ and calculated using eq 1, are shown in Figure 2. In the absence of salt, *G*_Index_ increased with temperature up to an optimum at 25–30 °C, after which it declined sharply at 35 °C. The addition of NaCl strongly inhibited root growth of *Triticum aestivum* at all temperatures, with no root development observed at 35 °C under saline conditions. As shown in Figure 2b, the results obtained by the students were in close agreement with those of the instructors, with only minor differences observed at 25–30 °C, leading to virtually identical conclusions.

**Figure 2:**
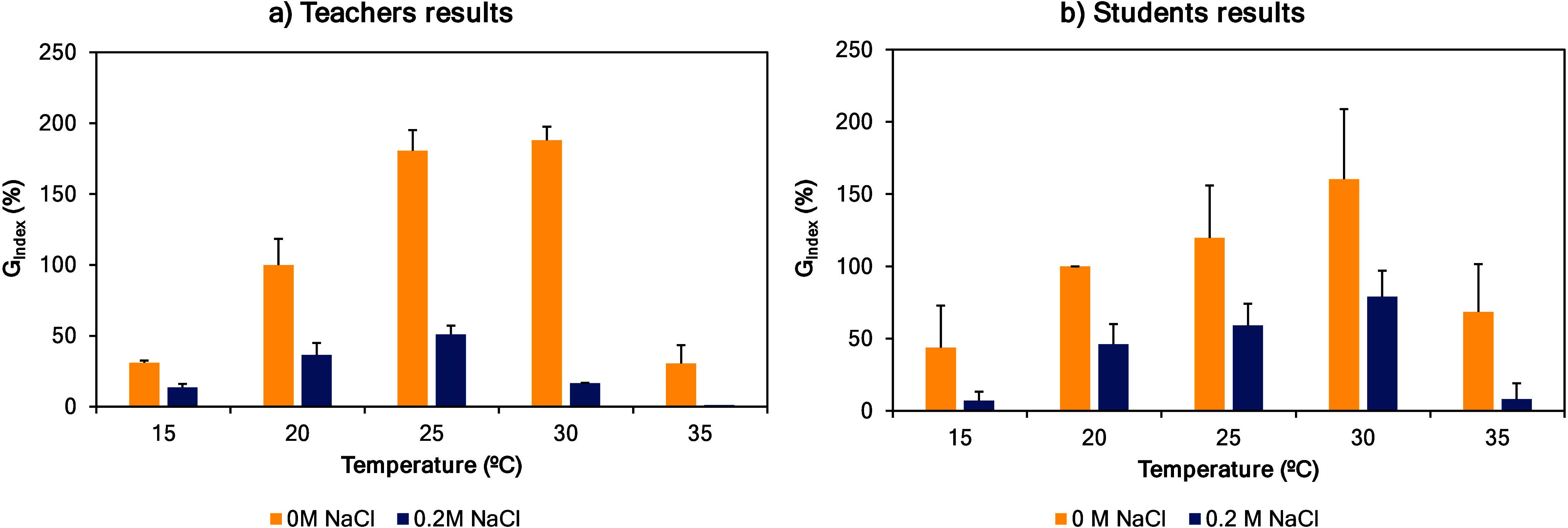
Root growth results of *Triticum aestivum* under different temperature (15, 20, 25, 30, 35 °C) and salinity conditions (0 M NaCl and 0.2 M NaCl), expressed as *G*_Index_. *G*_Index_ is a relative index normalized to the control conditions (20 °C, distilled water): (a) teachers’ results (*n* = 5) and (b) students’ results (*n* = 10). Error bars indicate 95% confidence intervals.

Shoot growth results, expressed as *P*_Index_ and calculated using eq 2, are shown in Figure 3. This pattern closely mirrored that observed for root growth: *P*_Index_ increased with temperature up to 25–30 °C, followed by a sharp decline at 35 °C. The presence of salt strongly inhibited shoot growth at all temperatures, with the most pronounced effects observed at 30–35 °C.

**Figure 3:**
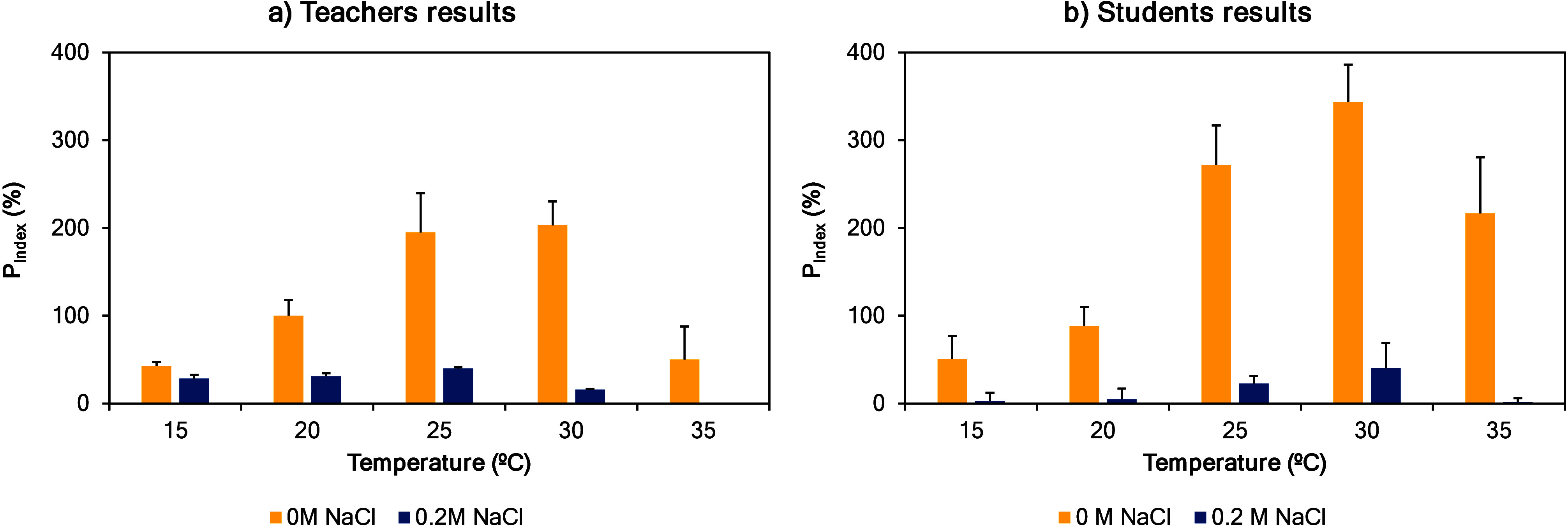
Shoot growth results of *Triticum aestivum* under different temperature (15, 20, 25, 30, 35 °C) and salinity conditions (0 M NaCl and 0.2 M NaCl), expressed as *P*_Index_. *P*_Index_ is a relative index normalized to the control condition (20 °C, distilled water): (a) teachers’ results (*n* = 5) and (b) students’ results (*n* = 10). Error bars indicate 95% confidence intervals.

The pronounced reductions in germination, root growth, and shoot development observed at 35 °C under saline conditions suggest that the combined effects of osmotic stress, ionic toxicity, and temperature-induced metabolic disturbances exceeded the physiological tolerance limits of wheat seedlings. These responses provide a basis for discussing the physiological and biochemical mechanisms underlying plant adaptation and responses to environmental stress. A discussion session was held between the instructors and the students (approximately 1 h) to review the results obtained and the possible underlying causes, as described below. Particular emphasis was placed on interpreting germination responses in terms of osmotic pressure, ionic strength, ion-specific effects, and changes in water availability under saline conditions. At temperatures above 25 °C and under a salinity of 0.2 M NaCl, *Triticum aestivum* exhibited progressively lower germination and seedling growth, with the strongest inhibitory effects observed at 35 °C. Under these conditions, the osmotic stress induced by the high salt concentration significantly reduces water uptake during imbibition,^9^ while the accumulation of Na^+^ and Cl^–^ in embryonic tissues increases ionic toxicity and disrupts the K^+^/Na^+^ balance, altering cellular homeostasis.^12^ In parallel, salinity induces overproduction of reactive oxygen species (ROS) and can compromise the antioxidant system, causing oxidative damage to lipids, proteins, and nucleic acids.^5^ Excessive ROS accumulation may lead to loss of membrane integrity, reduced enzymatic activity, and decreased metabolic efficiency during germination and early seedling development. This effect may be further intensified at elevated temperatures due to increased respiratory metabolism and greater membrane instability.

Moreover, salt stress promotes the accumulation of abscisic acid (ABA), a hormone associated with maintaining dormancy and stress response.^13^ Increased ABA inhibits the synthesis and action of gibberellins, limiting the induction of α-amylases in the aleurone layer.^14^ As a consequence, starch hydrolysis in the endosperm is reduced, decreasing the availability of soluble sugars needed for respiration and ATP production, which, in turn, affects cell division and elongation processes, delaying or even preventing radicle emergence.

During the discussion, six questions were posed regarding student learning, which were subsequently answered via an online platform (Movie) provided by our institution (University of Vigo). The students answered the questions through this platform, due to time constraints. Details of the questions are included in the Supporting Information (Subsection S2). The qualitative responses provided by students revealed a consistent and conceptually sound understanding of the effects of salinity and temperature on seed germination. A large majority of students (81% correct responses in Q1 and 73% in Q3; Table S4 and the Supporting Information) correctly identified the osmotic and ionic mechanisms underlying salinity stress, frequently referring to reduced water uptake, osmotic stress, and ion toxicity as key factors limiting germination and early plant growth, as evidenced by the representative student responses presented in the Supporting Information.

Students also demonstrated a clear understanding of temperature as a regulator of metabolic activity, with 85% of the respondents providing correct explanations of the effects of temperature on germination (Q2; Table S4 and the Supporting Information). Furthermore, several students explicitly referred to the combined effects of salinity and temperature, indicating their ability to integrate multiple environmental factors and interpret their interactions based on experimental observations. The concept of abiotic stress was generally well understood, with 73% of students providing correct definitions and examples, such as salinity, drought, and temperature extremes. In addition, students identified germination as a particularly sensitive developmental stage due to the lack of fully developed protective structures and their dependence on tightly regulated environmental conditions. Analysis of partially correct and incorrect responses revealed several recurring misconceptions. The most common difficulty involved distinguishing between osmotic stress and ion-specific toxicity, with some students attributing reduced germination mainly to the toxic effects of Na^+^ and Cl^–^ without explicitly considering the role of reduced water availability. Other students correctly identified salinity or temperature as stress factors but provided incomplete explanations of the underlying chemical or physiological mechanisms.

Beyond the direct observation of germination responses, this activity allows students to make explicit connections between experimental results and sustainable agricultural practices in the context of climate change. By analyzing the effects of salinity and temperature on early plant development, students gain an understanding of key processes such as osmotic stress, ionic toxicity, and altered water uptake, which are critical to crop yield in degraded or climate-affected soils. It is important to note that this understanding translates into agronomic reasoning. The goal is for students to relate their findings to real-world management strategies, including crop selection, optimization of irrigation practices, and soil management approaches aimed at mitigating the effects of salinity. In this way, the activity goes beyond descriptive experimentation and fosters systems-level thinking, where chemical and physiological processes are directly linked to sustainable decision-making in agriculture. Recent contributions in chemistry education have highlighted systems thinking as an effective framework for helping students integrate concepts across disciplinary boundaries and address complex sustainability-related challenges.^15,16^

Furthermore, by contextualizing these processes within climate change scenarios characterized by rising temperatures, students develop a more integrated perspective on the challenges facing modern agriculture and the role of chemistry in addressing them.

Although climate change is frequently associated with increased stress for crops, plant responses may vary, depending on species, developmental stage, and environmental conditions. For example, global-scale analyses have reported widespread vegetation greening associated with rising atmospheric CO_2_ concentrations, highlighting that climate change effects on plant growth are not universally negative.^17^ Beyond climate change, the activity is also relevant for discussing other agricultural and environmental challenges, including water scarcity, the use of saline or low-quality irrigation water, soil remediation and land reclamation, and the limitations imposed by abiotic stress on crop selection and geographical distribution.

Students may also compare the responses observed in wheat with those reported for other major crops. Wheat generally exhibits optimal germination and early growth at temperatures between 20 and 30 °C, whereas rice and maize typically perform best under slightly warmer conditions, commonly between 25 and 35 °C and 25 and 30 °C, respectively. In addition, rice tends to maintain growth and development at higher temperatures than wheat, whereas maize is generally considered more heat-tolerant during early developmental stages.^18^

From a pedagogical perspective, the responses highlight a successful integration of theoretical knowledge with experimental evidence, as students frequently referred to their direct observations (e.g., lack of germination under saline conditions) to support their explanations.

However, the qualitative analysis also revealed some limitations. Several students reported difficulties related to quantitative data processing, particularly the calculation and interpretation of *G*_Index_ and *P*_Index_ values, the normalization of root and shoot growth relative to the control treatment, and the comparison of responses among temperature and salinity treatments. Some students also found it challenging to interpret cases in which germination percentage and seedling growth showed different response patterns. These findings suggest that additional support in data processing and interpretation could further enhance learning outcomes. Students provided constructive feedback, suggesting improvements such as clearer explanations of calculations, inclusion of additional plant species, and extended incubation times. These insights can be valuable for refining the activity and improving its educational effectiveness in future implementations. Students’ learning was assessed through a combination of formative and summative approaches integrated within the course. Formative assessment included chemistry-oriented prelaboratory questions, classroom discussions, laboratory reports, and post-laboratory questionnaires, which provided continuous feedback on students’ understanding of salinity, temperature effects, and related chemistry concepts. Summative assessment was conducted through a final examination that included multiple-choice questions, short-answer questions, and problem-solving exercises based on the laboratory activities. The alignment between the proposed learning outcomes, assessment activities, and evidence of student learning is summarized in Table S5 of the Supporting Information.

During the laboratory sessions, particular attention was given to the explanation and discussion of data analysis and calculation procedures, ensuring that students understood how to process and interpret the experimental results. The explicit integration of chemistry-related concepts, curricular materials, assessment activities, and student evidence is further summarized in Table S6 of the Supporting Information.

At the end of the course, students were evaluated through a final examination that included a specific section dedicated to laboratory activities. This assessment combined multiple-choice questions, short-answer questions, and problem-solving exercises related to the practical sessions, including calculations performed during the experiments. These assessment activities provided additional evidence of students’ ability to interpret experimental results, apply chemistry-related concepts associated with salinity stress, and connect environmental stress factors with plant responses, consistent with the learning outcomes defined for the activity.

In addition, for the phytotoxicity assay, students were required to prepare a laboratory report presenting and interpreting the results described in this study, including germination indices and growth parameters. Furthermore, a post-laboratory questionnaire was administered to evaluate students’ conceptual understanding and their ability to relate experimental outcomes to climate change and agricultural contexts.

Student responses to the post-laboratory questionnaire were classified as correct, partially correct, incorrect, or no response according to predefined criteria aligned with the intended learning outcomes of the activity. Correct responses demonstrated an accurate understanding of the assessed concept, partially correct responses reflected incomplete but scientifically acceptable interpretations, and incorrect responses contained major conceptual errors. Between 69% and 85% of responses were classified as correct, while fewer than 10% were classified as incorrect. The distribution of responses is summarized in Table S4 of the Supporting Information.

Following the laboratory exercise, a satisfaction survey was conducted (34 students responded). Details of the survey are provided in the Supporting Information (Section S2). The results indicate that 85% of students were very satisfied, while 15% were satisfied.

## Conclusions

The conclusions, derived from the discussion between instructors and students, are summarized as follows:

(a) The negative effects of elevated temperature and salinity on germination become apparent only at 35 °C and a 0.2 M ionic strength.
(b) Root length of wheat increases with rising temperature, reaching a maximum between 25 and 30 °C; however, at temperatures above 30 °C, root growth decreases sharply. The presence of NaCl drastically reduces root development. Shoot growth follows a similar pattern to root growth, increasing with temperature up to a maximum and then declining sharply at high temperatures and in the presence of NaCl.
(c) Based on the students’ responses to the questions related to their learning, we can affirm that they have understood the basic concepts related to the effect of temperature and salinity on wheat germination.

## Supplementary Material



## Notes

Previous work conducted by the instructors, pre-laboratory activities, detailed methods, safety precautions, timetable, adaptation of the experiment, and post-laboratory activities (PDF)
