# Supplementary material for: Effects of Temperature and Salinity on the Germination of Triticum aestivum
Source: J Chem Educ. 2026 Jul 20;103(8):4590–7. doi: 10.1021/acs.jchemed.6c00286 (PMC13472044; doi:10.1021/acs.jchemed.6c00286)
Supplement: Supplementary file 1 [file ed6c00286_si_001.pdf]

SUPPORTING INFORMATION

Effects of Temperature and Salinity on the Germination of *Triticum aestivum*

Cecilia Martínez-Castillo[a], Manuel Arias-Estévez[a], Manuel Conde-Cid[a,b*], Daniel Arenas-Lago[a], Antía Gómez-Armesto[a,b]

[a]Department of Plant Biology and Soil Science, Faculty of Sciences, University of Vigo, As Lagoas S/N, 32004 Ourense, Spain

[b]REQUIMTE/LAQV, ISEP, Polytechnic of Porto, Rua Dr. António Bernardino de Almeida 431, 4249-015 Porto, Portugal

*Corresponding author: Manuel Conde-Cid (email: manconde@uvigo.es)

Table of contents

1. LABORATORY EXPERIMENT

1.1. Previous work made by the instructors

1.1.1. Soil

1.1.2. Germination Kits

1.2. Chemical background of the experiment

1.2.1. Chemistry-oriented pre-laboratory questions

1.3. Pre-Laboratory activities

1.3.1. Pre-Laboratory Questions

1.4. Laboratory activities

1.4.1. Safety

1.4.2. Apparatus and other laboratory materials

1.4.3. Chemicals required

1.5. Timetable

1.6. Adaptation of the experiment

1.7. Estimated cost of the laboratory activity

1.7.1. Cost per Student

2. POST-LABORATORY ACTIVITIES

3. ALIGNMENT OF LEARNING OUTCOMES, ASSESSMENT, AND EVIDENCE

4. INTEGRATION OF CHEMISTRY CONCEPTS WITH CURRICULAR ACTIVITIES

1. LABORATORY EXPERIMENT

1.1. Previous work made by the instructors

1.1.1. Soil

After collection, the soil sample was air-dried and sieved. The fraction smaller than 2 mm was used for the experiment and stored in plastic containers until distributed to the students. The methods employed to determine the soil's physicochemical properties, including pH, organic matter content, particle size distribution, and effective cation exchange capacity, are described in detail in Bigham (1996).

1.1.2. Germination Kits

The Phytotoxkit microbiotest includes all materials required to perform a complete phytotoxicity assay. In this study, wheat (*Triticum aestivum*) seeds were used. Wheat is widely cultivated for human consumption and is a monocotyledonous species with relatively high tolerance to salinity. Its rapid germination enables observations to be conducted within three days of test initiation.

The assays were performed in transparent test plates that allow direct observation of germination and measurement of root and shoot length for each seedling through image analysis at the end of the exposure period. The kit provides clear instructions and detailed illustrations to facilitate standardized test procedures. All experiments were conducted in triplicate.

Compared with conventional phytotoxicity assays, this microbiotest ensures high repeatability and reproducibility. The characteristics and methodological details of the test have been described previously in Santas-Miguel et al. (2022).

1.2. Chemical background of the experiment

The phytotoxicity assay was designed not only to evaluate plant responses to environmental stress, but also to help students apply fundamental chemistry concepts to a biologically relevant system.

The chemistry concepts and questions presented in this section reflect the content discussed with students before and during the laboratory activity. To improve transparency, reproducibility, and usefulness for instructors, some explanatory details have been expanded in this Supporting Information relative to the materials originally provided to students.

In this experiment, salinity stress was simulated using a 0.2 M NaCl solution. The addition of NaCl increases the concentration of dissolved ions (Na^+ and Cl^-) in the soil solution, thereby increasing ionic strength and osmotic pressure. Under these conditions, seeds experience reduced water availability during imbibition, which negatively affects germination and early seedling growth.

Students were introduced to the concept that water movement into seeds depends on differences in water potential between the seed tissues and the surrounding medium. Increasing solute concentration in the external solution reduces water potential and limits water uptake.

The osmotic pressure generated by the NaCl solution can be approximated using the van ’t Hoff equation:

$$\Pi = iCRT$$

where Π is osmotic pressure, i is the van ’t Hoff factor, C is molar concentration, R is the gas constant, and T is temperature in Kelvin.

For NaCl solutions, the dissociation into Na^+ and Cl^- ions increases the number of dissolved particles in solution, thereby increasing osmotic effects. Students were encouraged to relate this increase in osmotic pressure to the reduced germination and root growth observed experimentally.

In addition, the experiment introduced students to the concepts of ionic toxicity and ionic imbalance. The dissociation of NaCl into Na^+ and Cl^- ions increases the number of dissolved particles in solution, thereby increasing osmotic effects. As a consequence, the chemical potential of water decreases, reducing water availability for seeds during imbibition. Students were encouraged to relate this process to the experimentally observed reductions in germination and root growth. Furthermore, elevated Na^+ concentrations may interfere with nutrient uptake and disrupt cellular homeostasis, contributing to impaired seedling development under saline conditions.

These concepts were discussed qualitatively during the laboratory sessions and were connected to soil chemical properties previously studied in the course, including soil pH, cation exchange capacity, exchangeable cations, and soil salinity.

1.2.1. Chemistry-oriented pre-laboratory questions

1) How does NaCl dissociation modify the number of dissolved particles in the soil solution?
2) Explain how increasing NaCl concentration may reduce water uptake during seed imbibition.
3) Using the van ’t Hoff equation, explain qualitatively why osmotic pressure increases in saline solutions.
4) How may high concentrations of Na^+ interfere with nutrient uptake and ionic balance in plants?
5) Why can soil chemical properties such as pH and cation exchange capacity influence plant responses to salinity?

1.3. Pre-Laboratory activities

Before the lab session, students were provided with preparatory materials designed to introduce theoretical background and help them understand the experiment.

The preparatory materials described below summarize the information provided to students and include additional explanatory details incorporated in the revised Supporting Information for instructional purposes.

These materials included a manual of seed germination processes and the influence of environmental factors such as temperature and salinity on plant development. In particular, key chemical concepts such as osmotic potential, ionic strength, and ion toxicity were introduced and explicitly linked to the experimental conditions used in this activity, including the application of NaCl solutions and different incubation temperatures.

The teachers conducted preliminary tests on the growth of wheat roots and shoots, including germination at different NaCl concentrations, in order to select the most useful saline concentration for educational purposes. The results are shown in Table S1.

Table S1. Mean shoot and root growth of wheat (mm ± SD) at different NaCl concentrations at 25 °C.

NaCl concentration (M)	Shoot growth (mm)	Root growth (mm)
0.00	19.78 ± 4.65	73.21 ± 7.95
0.01	12.95 ± 3.34	59.72 ± 4.59
0.10	5.82 ± 3.16	38.57 ± 7.68
0.20	0.91 ± 0.41	8.36 ± 1.23

In addition, students were given a set of guiding questions to encourage critical thinking and to help them anticipate how temperature and salinity could affect water uptake, germination, and early seedling growth. These questions were intended to promote the integration of theoretical knowledge with experimental observations.

These materials were intended to provide students with the necessary conceptual framework to interpret the experimental results and to relate them to broader environmental and agricultural challenges.

1.3.1. Pre-Laboratory Questions

1) What is osmotic stress and how does it affect water uptake in seeds?

2) How can the presence of NaCl influence ionic balance in plant tissues?

3) Why does temperature influence the germination rate of seeds?

1.4. Laboratory activities

1.4.1. Safety

Students were required to follow standard laboratory safety and hygiene practices. Personal protective equipment (PPE), including laboratory coats, safety glasses, and gloves, was mandatory during all experimental steps.

Soil was handled using spatulas and other tools to avoid direct contact. Although the soil was air-dried and sieved, it was not sterilized and may contain naturally occurring microorganisms; therefore, hand hygiene was required after handling.

To avoid cross-contamination, all materials in contact with soil or solutions were cleaned with water between treatments and work surfaces were kept clean during the experiment. Dust generation during soil weighing and transfer was minimized.

Waste soil, seeds, and plant residues were collected and disposed of as non-hazardous laboratory waste according to institutional guidelines.

1.4.2. Apparatus and other laboratory materials

- Germination kit (Phytotoxkit)
- Small incubators
- Camera or flatbed scanner
- Image analysis software (ImageJ)
- Measuring spoons
- Analytical balance
- 100 mL beakers
- Spatula
- Graduated cylinders
- Bottles containing soil solutions (500 and 50 mg kg^{-1})
- Wash bottles

- Rulers
- Forceps

1.4.3. Chemicals required

The reagent used in this laboratory exercise, sodium chloride (NaCl), was supplied by PANREAC (Barcelona, Spain). Key information regarding the reagent is presented in Table S2.

Table S2. Characteristics of the reagent used in the experiment.

Reagent	CAS No.	Purity	MW (g/mol)	Form
NaCl	7647-14-5	≥99.0%	58.44	Crystalline solid

MW: molecular weight.

1.5. Timetable

The total duration of the students' exercise is approximately 8 hours. The activities are divided into two phases, which will be carried out on separate days, with their respective approximate durations as follows:

Phase 1

This phase takes approximately 3 hours and 30 minutes.

Task 1. Introduction to the exercise by the instructor – 30 min

Task 2. Calculation of the water volume required to reach field capacity for the soils – 30 min

Task 3. Weighing soil for the plates, depositing, and leveling (3 plates) – 30 min

Task 4. Adding solutions evenly and placing filters (10 plates) – 30 min

Task 5. Placing seeds and closing plates (10 plates) – 30 min

Note: The seeds were incubated in the dark in a climatic chamber for 72 hours.

Phase 2

This phase takes approximately 4 hours and 30 minutes.

Task 6. Assessment of germination, root, and shoot growth (3 plates) – 150 min

Task 7. Cleaning – 45 min

Task 8. Group discussion on technical issues – 60 min

Task 9. Completion of the satisfaction survey – 15 min

1.6. Adaptation of the experiment

When conducting this laboratory experiment in other countries, two considerations should be taken into account:

a) The soil used can be of any type; however, its physicochemical properties should always be determined. It is recommended to use nutrient-poor soils to avoid interference with seed germination.

b) Germination kits are generally easy to obtain and relatively inexpensive. Additional information is available at https://ecotestsl.com/producto/phytotoxkit/. If budgetary constraints prevent the use of these kits, germination experiments can alternatively be performed using much less expensive Petri dishes.

1.7. Estimated cost of the laboratory activity

The estimated cost of the laboratory activity was calculated based on the materials required to perform the germination assays using the Phytotoxkit system and standard laboratory supplies. Table S3 summarizes the approximate costs associated with the main components of the experiment.

Table S3. Estimated cost of materials used in the laboratory activity (n = 34 students).

**Item**	**Unit cost (€)**	**Quantity used**	**Total cost (€)**	**Notes**
Phytotoxkit (germination kits)	120–150	4–5 kits	480–600	Main cost component
Sodium chloride (NaCl)	<1	1	<1	Negligible cost
Filter paper / consumables	~10–15	1	10–15	Includes minor materials
Water and basic lab supplies	negligible	—	negligible	Not accounted
Total estimated cost	—	—	~500–600	—

1.7.1. Cost per Student

A total of 34 students participated in the laboratory activity, working in groups of three and preparing a total of 120 experimental plates. Based on the total estimated cost (~€500–600), the cost per student was approximately €15–20.

2. POST-LABORATORY ACTIVITIES

Students are required to prepare a laboratory report that includes measurements of root and shoot length, as well as calculations of various germination indices. The report should also include responses to the following questions:

1) Do you understand how salinity can affect seed germination and early plant growth?
2) Can you describe how temperature influences seed germination?
3) Do you understand what is meant by abiotic stress in plants? Please provide two examples.
4) Can you identify two environmental factors that may have a combined effect on plants?
5) Why is germination a particularly sensitive stage to environmental stress factors?
6) Which concept related to germination or abiotic stress did you better understand through this activity?

Examples of anonymized student responses illustrating students' ability to connect experimental observations with chemistry-related concepts are presented below.

Q1. "NaCl dissociates into Na^+ and Cl^- ions, increasing osmotic pressure and reducing water uptake during seed imbibition, which limits germination."/ "High salt concentrations create osmotic stress and negatively affect root development."

Q2. "Temperature regulates metabolic and enzymatic activity during germination."/ "Very low or very high temperatures inhibit seed development and growth."

Q3. "Abiotic stress is caused by non-living environmental factors such as salinity or heat."/ "High Na^+ concentrations may disrupt ionic balance and interfere with nutrient uptake, reducing plant growth."

Q4. "High salinity and high temperature together strongly reduce germination."/ "Temperature and water availability interact and affect plant growth simultaneously."

Q5. "Seeds strongly depend on water uptake during germination."/ "Environmental stress can inhibit enzymatic activity and root development."

Q6. "I better understood how osmotic pressure affects water absorption."/ "The activity helped me understand how salinity changes the chemical environment surrounding the seed."

Student responses to the post-laboratory questionnaire were classified as correct, partially correct, incorrect, or no response according to predefined criteria aligned with the intended learning outcomes. A summary of student performance is provided in Table S4.

Table S4. Summary of student performance in the post-laboratory questionnaire.

**Question**	**Correct (%)**	**Partially correct (%)**	**Incorrect (%)**	**No response (%)**
Q1 - Salinity and germination	81	15	4	0
Q2 - Temperature effects	85	11	4	0
Q3 - Abiotic stress	73	19	4	4
Q4 - Environmental factors	69	19	8	4
Q5 - Sensitivity of germination	73	15	9	3
Q6 - Concept understood	77	12	8	3

Finally, students completed a satisfaction survey, the results of which are discussed in the manuscript. The survey employed a four-point rating scale:

1. Very satisfied
2. Satisfied
3. Dissatisfied
4. Very dissatisfied

The final examination assessed the broader learning outcomes of the Soil Science course rather than this laboratory activity specifically. Nevertheless, several examination questions addressed concepts closely related to the experiment, including soil chemistry, nutrient retention, soil–plant interactions, and environmental stress.

Representative examples included:

- What effect does an acidic pH have on the mobility of certain elements in the soil?
- How can soil contribute to climate regulation?
- What is the main objective of measuring cation exchange capacity (CEC) in an agricultural soil?
- How does increasing NaCl concentration affect water uptake during seed germination?

- Why can high salinity reduce root and shoot growth in germinating seedlings?
- How can temperature modify the effects of salinity stress on seed germination and early plant development?

These questions show how concepts studied during the laboratory activity were reinforced within the general assessment framework of the Soil Science course.

3. ALIGNMENT OF LEARNING OUTCOMES, ASSESSMENT, AND EVIDENCE

Table S5. Alignment between learning outcomes, assessment activities, and evidence of student learning.

Learning outcome	Assessment activity	Evidence/artifact
Explain how salinity modifies the chemical environment through ionic strength and osmotic pressure	Chemistry-oriented pre-laboratory questions and post-laboratory discussion	Student responses Q1 and Q6
Relate osmotic pressure and water availability to seed germination and seedling growth	Laboratory report and germination index calculations	Experimental data interpretation and growth indices
Interpret the role of Na^+ and Cl^- in ionic imbalance and salinity stress	Post-laboratory questions and classroom discussion	Student responses Q1, Q3, and Q4
Connect soil chemical properties with plant responses	Integration with soil laboratory sessions (pH, CEC, exchangeable cations)	Laboratory discussions and comparative interpretation
Interpret quantitative experimental results	Calculation of germination and growth parameters	Laboratory reports and calculation exercises
Communicate scientific findings related to salinity and climate change	Laboratory report and qualitative questionnaire	Written discussions and anonymized student responses

4. INTEGRATION OF CHEMISTRY CONCEPTS WITH CURRICULAR ACTIVITIES

Table S6. Chemistry concepts addressed through the activity and their integration into curricular resources and assessment.

**Chemistry concept**	**Curricular resource**	**Assessment activity**	**Evidence**
Osmotic pressure	Chemical Background section	Pre-lab Questions 2–3	Student responses Q1 and Q6
Ionic strength	Chemical Background section	Pre-lab Question 1	Student responses Q1
Ionic toxicity	Chemical Background section	Pre-lab Question 4	Student responses Q1 and Q3
Water potential	Chemical Background section	Post-laboratory discussion	Student responses Q5
Soil chemical properties (pH, CEC, exchangeable cations)	Soil laboratory sessions	Comparative interpretation and report discussion	Laboratory reports and classroom discussion

Table S7. Integration of soil chemistry laboratory activities.

**Laboratory activity**	**Main chemistry concepts**	**Learning outcome**	**Evidence**
Soil pH determination	Acid-base chemistry	Interpret soil acidity and nutrient availability	Laboratory report
Organic matter determination	Organic matter and soil fertility	Relate OM to soil quality	Laboratory report
Cation exchange capacity	Surface charge and ion exchange	Understand nutrient retention processes	Calculation exercises
Exchangeable cations	Soil solution chemistry	Interpret nutrient availability	Laboratory report

Laboratory activity	Main chemistry concepts	Learning outcome	Evidence
Phytotoxicity assay	Osmotic pressure, ionic strength, ion toxicity	Relate soil chemistry to plant responses	Report + questionnaire

REFERENCES

Bigham, J. M., Ed. *Methods of Soil Analysis: Part 3. Chemical Methods*; Soil Science Society of America: Madison, WI, **1996**.

Santás-Miguel, V.; Arias-Estévez, M.; Arenas-Lago, D. Phytotoxicity of Heavy Metals in Two Acid Soils with Different Organic Matter: The Case of Zn. *J. Chem. Educ.* **2022**, 99, 3272-3276. https://doi.org/10.1021/acs.jchemed.2c00520.
